# A Cross-Sectional Evaluation of the Virtual Outpatient Management of People With Mpox

**DOI:** 10.1093/ofid/ofae413

**Published:** 2024-07-26

**Authors:** Clare E Warrell, Zain Chaudhry, Marianne Shawe-Taylor, Evanthia Mastoraki, Ashwin Delmonte Sen, Hannah Rafferty, Angus De Wilton, Naomi Mescall, Catherine Houlihan, Philip Gothard, Eva Jungmann, Sarah Logan, Tommy Rampling, Laura Waters, Rita Browne, Michael Marks, Emily Shaw

**Affiliations:** Division of Infection, Hospital for Tropical Diseases, University College London Hospitals NHS Foundation Trust, London, United Kingdom; Rare and Imported Pathogens Laboratory, UK Health Security Agency, Wiltshire, United Kingdom; Division of Infection, Hospital for Tropical Diseases, University College London Hospitals NHS Foundation Trust, London, United Kingdom; Division of Infection and Immunity, University College London, London, United Kingdom; Division of Infection, Hospital for Tropical Diseases, University College London Hospitals NHS Foundation Trust, London, United Kingdom; Department of Sexual Health and HIV, Central and North West London NHS Trust, London, United Kingdom; Division of Infection, Hospital for Tropical Diseases, University College London Hospitals NHS Foundation Trust, London, United Kingdom; Division of Infection, Hospital for Tropical Diseases, University College London Hospitals NHS Foundation Trust, London, United Kingdom; Division of Infection, Hospital for Tropical Diseases, University College London Hospitals NHS Foundation Trust, London, United Kingdom; Division of Infection, Hospital for Tropical Diseases, University College London Hospitals NHS Foundation Trust, London, United Kingdom; Rare and Imported Pathogens Laboratory, UK Health Security Agency, Wiltshire, United Kingdom; Division of Infection and Immunity, University College London, London, United Kingdom; Department of Clinical Virology, University College London Hospitals NHS Foundation Trust, London, United Kingdom; Division of Infection, Hospital for Tropical Diseases, University College London Hospitals NHS Foundation Trust, London, United Kingdom; Department of Sexual Health and HIV, Central and North West London NHS Trust, London, United Kingdom; Division of Infection, Hospital for Tropical Diseases, University College London Hospitals NHS Foundation Trust, London, United Kingdom; Division of Infection, Hospital for Tropical Diseases, University College London Hospitals NHS Foundation Trust, London, United Kingdom; Rare and Imported Pathogens Laboratory, UK Health Security Agency, Wiltshire, United Kingdom; Division of Infection and Immunity, University College London, London, United Kingdom; National Institute for Health and Care Research, University College London Hospitals Biomedical Research Centre, London, United Kingdom; Department of Sexual Health and HIV, Central and North West London NHS Trust, London, United Kingdom; Department of Sexual Health and HIV, Central and North West London NHS Trust, London, United Kingdom; Division of Infection, Hospital for Tropical Diseases, University College London Hospitals NHS Foundation Trust, London, United Kingdom; Division of Infection and Immunity, University College London, London, United Kingdom; Clinical Research Department, London School of Hygiene and Tropical Medicine, London, United Kingdom; Division of Infection, Hospital for Tropical Diseases, University College London Hospitals NHS Foundation Trust, London, United Kingdom

**Keywords:** monkeypox virus, mpox, virtual ward, high consequence infectious disease (HCID), telemedicine

## Abstract

**Background:**

To report on the implementation and outcomes of a virtual ward established for the management of mpox during the 2022 outbreak, we conducted a 2-center, observational, cross-sectional study over a 3-month period.

**Methods:**

All patients aged ≥17 years with laboratory polymerase chain reaction–confirmed monkeypox virus managed between 14 May and 15 August 2022, at the Hospital for Tropical Diseases at University College London Hospitals National Health Service (NHS) Foundation Trust and sexual health services at Central North and West London NHS Foundation Trust, were included. Main outcomes included the proportion of patients managed exclusively on the virtual ward, proportion of patients requiring inpatient admission, proportion of patients with human immunodeficiency virus, and duration of lesion reepithelialization.

**Results:**

Among confirmed cases (N = 221), 86% (191/221) were managed exclusively on the virtual ward, while 14% (30/221) required admission. Treatment for concomitant sexually transmitted infections was provided to 25% (55/221) of patients, antibiotics for other infective complications to 16% (35/221), and symptomatic relief to 27% (60/221). The median time from onset to complete lesion reepithelialization and de-isolation was 18 days (range, 8–56 days). Eleven percent (24/221) of individuals disengaged from services within 4 days of testing.

**Conclusions:**

The virtual ward model facilitated safe and holistic outpatient management of mpox, while minimizing admissions. This approach could serve as a model for future outbreak responses.

## Teaser

We assessed a mpox virtual outpatient model during the 2022 outbreak, when it was classified as a high consequence infectious disease (HCID). 86% patients were managed virtually, reducing admissions. This model could inform future HCID responses, enabling safe remote care.

2022 heralded an unprecedented, worldwide outbreak of mpox (formerly known as human monkeypox), featuring previously undocumented sustained person-to-person transmission of monkeypox virus (MPXV), primarily via sexual or close contact [1]. The majority of cases have been of clade IIb B.1 and sublineages [2]. From May 2022, locally acquired cases were reported from England and other nonendemic countries [3–5]. In July 2022, the World Health Organization (WHO) declared mpox a public health emergency of international concern, and by the end of the year, there had been 3582 laboratory-confirmed cases of mpox in the United Kingdom (UK) and 83 974 from WHO Member States [6–8].

At the onset of the UK outbreak, mpox was considered a high-consequence infectious disease (HCID) based on the previous case-fatality data from West and Central Africa, the absence of clinically proven efficacious vaccines or treatment, and its transmissibility in community and healthcare settings [9–12]. Therefore, the standard of care at the time was admission to dedicated specialist inpatient units, coordinated via a national HCID network [13].

During the epidemic, case numbers in London grew rapidly. The first confirmed case was on 6 May 2022, and by 8 June there were >300 confirmed cases, overwhelming HCID inpatient capacity [14]. Early reports highlighted a lower case-fatality rate in the 2022 outbreak than previously reported from Africa [15, 16]. The rapid rise in case numbers, combined with the lower observed case-fatality rate, suggested that admission of all cases was neither feasible, nor necessary.

Virtual wards have been successfully utilized for a variety of other conditions including coronavirus disease 2019 [17]. In May 2022, the University College London Hospital (UCLH) Hospital for Tropical Diseases (HTD) and sexual health services at Central North and West London (CNWL) were among the first centers nationally to establish a holistic, outpatient, integrated care pathway for individuals with suspected and confirmed mpox. UK outpatient management of HCIDs was unprecedented to this point. Central to this was a virtual ward, used to deliver care to the majority of individuals with mpox in our catchment area. Development of the pathway was informed by guidance produced by the UK Health Security Agency (UKHSA) and Nigeria Centre for Disease Control, in addition to publications from endemic regions, reflective of the latter’s extensive experience of mpox case management [18–20]. Herein, we report cases of mpox managed via the virtual ward service and describe its operational organization.

## PARTICIPANTS AND METHODS

We conducted a 2-center, observational, consecutive, case note review of all patients aged ≥17 years with laboratory polymerase chain reaction (PCR)–confirmed MPXV who received their care during a 3-month period from 14 May to 15 August 2022 at HTD and CNWL.

### Diagnosis of Mpox

Adults self-presenting or referred to UCLH emergency department or CNWL sexual health services who met the UKHSA criteria as a probable case of mpox (unexplained rash and a sexual, travel, or contact risk factor) were isolated in dedicated clinical areas and assessed and tested by trained clinicians wearing suitable personal protective equipment [21]. Swabs from lesions, the throat, and/or rectum were tested for MPXV and orthopoxvirus DNA by real-time PCR, performed at the UKHSA Rare and Imported Pathogen Laboratory. Tests for sexually transmitted infections (STIs), routine blood tests, and imaging were performed as clinically indicated, with samples processed locally.

### Risk Assessment

Individuals with probable mpox who were likely part of the UK-circulating outbreak were assigned to a National Health Service (NHS)–defined risk group, determined by their clinical status and ability to safely isolate in the community [22]. Those with a clinical indication such as severe clinical illness, complications or at heightened risk of complications (risk group A), or who were unable to safely isolate in the community (risk group B) were admitted, coordinated by the national HCID network [23]. At discharge, these patients were transferred to the virtual ward.

**Figure 1.**
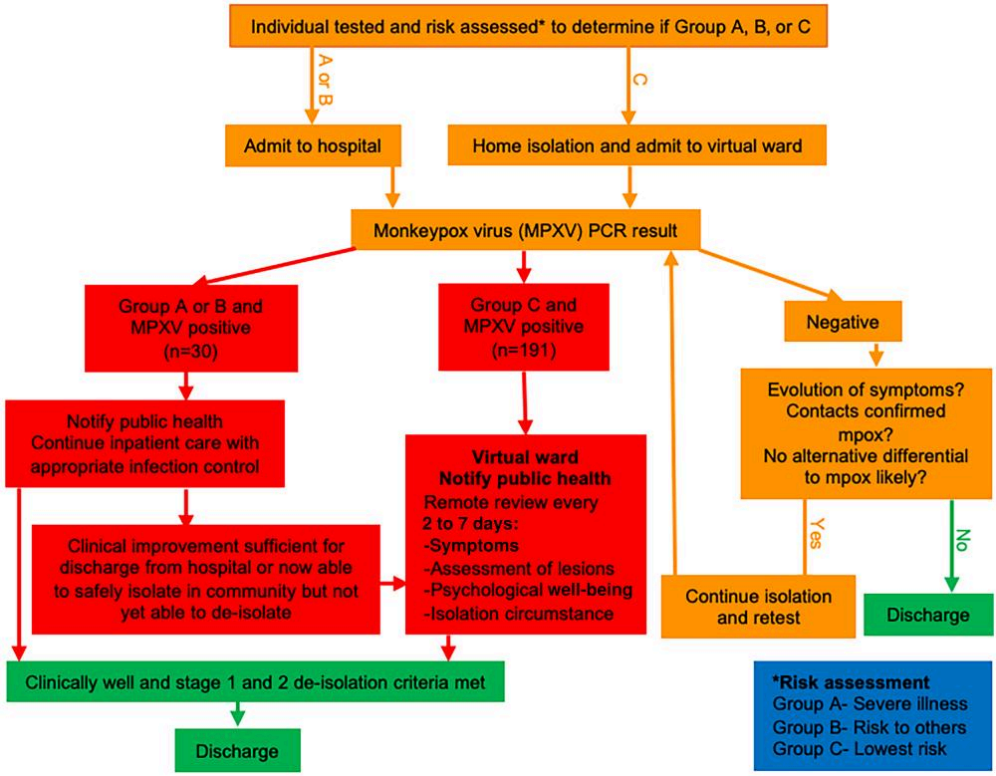
Standardized care pathway for management of individuals with suspected mpox. Abbreviations: MPXV, monkeypox virus; PCR, polymerase chain reaction.

Individuals who were assessed as clinically stable and able to safely isolate in the community pending their results (risk group C) were enrolled onto the virtual ward and managed using a standardized care pathway (Figure 1). Individuals were advised to walk home wearing a surgical face mask and with all lesions covered with clothing or dressings and asked to follow UKHSA isolation guidelines [18]. Individuals were advised about the potential mental health impact of isolation and how to seek psychological and financial support.

### Virtual Ward Management

Outpatients whose initial MPXV PCR testing was reported as negative, but for whom there continued to be clinical suspicion of mpox, were advised to continue self-isolation and were invited for retesting. These patients predominantly fell into 3 groups: (1) presence of a suspicious lesion(s), without a satisfactory alternative diagnosis, (2) evolution of symptoms, or (3) new information regarding a confirmed-positive contact. Individuals with a negative MPXV PCR and a satisfactory alternative diagnosis were discharged with appropriate treatment and follow-up was arranged.

Individuals with confirmed MPXV were informed of their result and notified to the health protection authority, the HCID network (where consent was granted), the individual's general practitioner, and (if relevant) their human immunodeficiency virus (HIV) team. While enrolled on the virtual ward, telephone consultations were undertaken by suitably trained and supervised infectious diseases physicians or senior nurses up to every 48 hours. In addition, a dedicated advice line/email address was established to allow patient-initiated contact.

#### Clinical Assessment

Standardized consultation templates were utilized. At each appointment, the individual's physical well-being was assessed including the evolution of their lesions, aided (where individuals opted to) by up-to-date photographs sent to a secure email inbox in advance of each consultation, and enquiries pertaining to any potential complications of mpox. Mental well-being was included as part of a comprehensive assessment. Patients were provided with the details of a dedicated mpox phone, held by an infectious diseases registrar, for any queries or concerns.

#### Medications

At each appointment, newly available test results inclusive from STI screening were relayed and, where required, prescription medication was dispatched from our pharmacies to a home address or individuals were invited to return for a dose of parenteral antibiotics (if not already administered empirically on the initial assessment).

#### De-isolation

**Figure 2.**
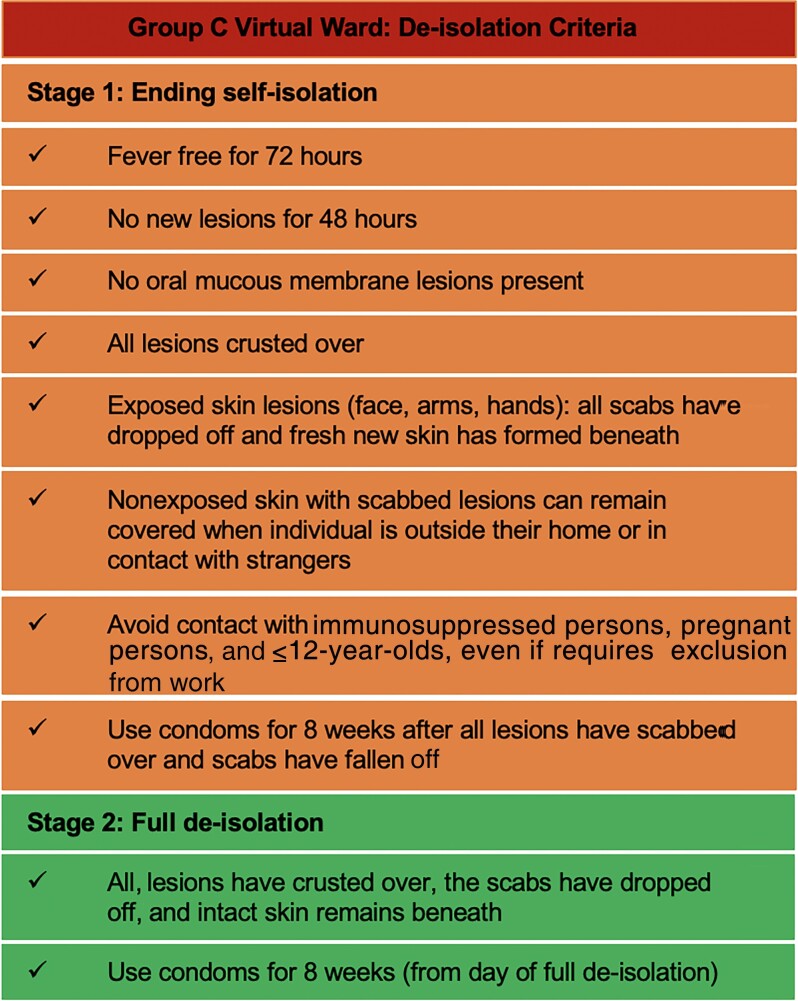
De-isolation criteria for individuals isolating at home with confirmed mpox [22, 24].

An integrated approach using both patient-submitted photographs and telephone assessment facilitated de-isolation decisions using a 2-stage de-isolation policy as per the UKHSA guidance (Figure 2) [22, 24]. Full de-isolation could occur when all lesions had reepithelialized [24]. During each assessment, individuals were either advised to continue to self-isolate or to proceed to the next stage of de-isolation. Individuals working in professions with contact with potentially vulnerable populations were asked to liaise with their health protection authority and their occupational health department for guidance regarding their return to work. Repeat viral PCR swabs for de-isolation purposes were not performed routinely by our services.

### Data Extraction

All consecutive patients aged ≥17 years with laboratory-confirmed MPXV tested between 14 May and 15 August 2022 and managed by our virtual ward service were included in the analysis. Electronic hospital records including standardized case reports of all participants were retrospectively interrogated for data extraction inclusive of demographics, epidemiological details (sexual, travel, and contact histories), smallpox/mpox vaccination history, HIV status, clinical features (relating to lesions, systemic symptoms, and complications), dates of remote consultations, concomitant STIs, and medications supplied. Clinical outcomes were followed up to 15 September 2022.

The incubation period was defined as the interval between the date of contact with the most likely source of transmission (a person described as having classic lesions, a person who subsequently informed them they had confirmed mpox or their only recent sexual contact) and first symptom onset. Individuals in whom the date of inoculation was unclear were excluded from the incubation period estimate.

For the purpose of investigating possible route of inoculation, sexually active gay, bisexual, and other men who have sex with men (GBMSM) were differentiated into those who reported receptive but not insertive anal sex (with or without oral or vaginal insertive sex); insertive but not receptive anal sex (with or without oral or vaginal insertive sex); insertive and receptive anal sex (with or without oral or vaginal insertive sex); and oral sex only.

### Analysis

Continuous variables are presented as medians and ranges, and categorical variables as absolute values and percentages (the latter given using the complete denominator, inclusive of where there were missing data). Continuous variables were compared using a 2-tailed *t* test and a significance threshold of .05. All analyses were performed in Prism software (version 9.4.1).

## RESULTS

### Demographics

Between 14 May and 15 August 2022, 497 individuals in total were tested for MPXV at HTD and CNWL, and 221 individuals (44% of those tested) had PCR-confirmed MPXV. The demographic and epidemiological characteristics of the confirmed cases are detailed in Table 1. The median age was 38 years (range, 17–65 years), 219 individuals (99%) identified as male, and 211 (95%) identified as GBMSM. Two individuals (1%) denied any sexual contact in the 3 months prior to onset of symptoms and 178 (81%) were documented as having at least 1 casual sexual partner in the prior 3 months. Thirty-four individuals (15%) reported sexual or household contact with a confirmed case of mpox; 34 (15%) reported travel outside of UK in the 3 weeks prior to onset of symptoms, with the majority traveling to Spain and/or Germany and none reporting travel to mainland West or Central Africa. Apart from 1 individual who had received the smallpox vaccine in 1980, none had been vaccinated against mpox outside of the incubation period. Eighty-eight (40%) were persons with HIV (PWH), 1 of whom was newly diagnosed. The majority of PWH (73/88 [82% of those HIV positive]) were known to be virally suppressed on antiretroviral therapy (ART) at the point of mpox diagnosis.

**Table 1.** Demographics and Epidemiological Characteristics of the Individuals With Confirmed Mpox

Characteristic	Individuals (N = 221)
Age, y, median (range)	38 (17–65)
Sex	
Male	219 (99)
Female	1 (<1)
Transgender man without sex reassignment surgery	1 (<1)
Ethnicity	
White British or Irish	61 (28)
Another White background	59 (27)
Black African, Black Caribbean, or Black other	13 (6)
Chinese	6 (3)
Other	26 (12)
Missing data	56 (25)
Sexual orientation	
GBMSM	211 (95)
Heterosexual men	3 (1)
Not yet sexually active	1 (<1)
Missing data	6 (3)
Vaccination status	
Mpox vaccine naive	210 (95)
First dose of mpox vaccine same day/after developed symptoms	6 (3)
First dose of mpox vaccine prior to symptom onset	4 (2)
Smallpox vaccinated	1 (<1)
HIV status	
HIV negative	128 (58)
HIV status unknown	5 (2)
HIV negative/unknown and currently taking PrEP	68 (31)
HIV positive	88 (40)
Well controlled on ARVs with undetectable VL	73 (33)
With CD4 <200 cells/μL, VL >50 copies/mL, poor ARV adherence, or new diagnosis	8 (4)
Further data missing	7 (3)
Risk factors	
≥1 casual sexual partner in last 3 mo	178 (81)
Denied casual sexual partners in last 3 mo	5 (2)
Missing data regarding casual sexual partners in last 3 mo	38 (17)
Sexual or household contact with confirmed mpox	34 (15)
Recreational drugs during sex/sex parties/licensed sex premise	40 (18)
Sex work/adult film industry	4 (2)
Sex with a sex worker	1 (<1)
Travel outside of UK in last 3 wk	34 (15)
Type of sex	
Anal receptive	12 (5)
Anal insertive	8 (4)
Oral	11 (5)
Vaginal insertive	5 (2)
Anal receptive and oral	19 (9)
Anal insertive and oral	26 (12)
Anal receptive and insertive	11 (5)
Anal receptive, insertive, and oral	58 (26)
Anal receptive, insertive, vaginal insertive, and oral	1 (0)
Anal insertive, vaginal insertive, and oral	5 (2)
Anal receptive, vaginal insertive, and oral	2 (1)
Vaginal receptive and oral	1 (0)
Missing data	62 (28)

Data are presented as No. (%) unless otherwise indicated.

Abbreviations: ARV, antiretroviral; GBMSM, gay, bisexual, or other men who have sex with men; HIV, human immunodeficiency virus; PrEP, preexposure prophylaxis; UK, United Kingdom; VL, viral load.

Six individuals (3%) were MPXV PCR negative on initial testing at our centers but were recalled for repeat testing (2–13 days after their first swabs) and subsequently tested positive. Three individuals were MPXV PCR negative on initial testing, were recalled on account of a high clinical index of suspicion of mpox, and were again negative for MPXV on repeat testing (3–7 days after their first swabs). Thirty-five individuals (16%) with PCR-confirmed MPXV reported that they had been seen on 1–3 separate occasions by other health providers prior to accessing our services for the same symptoms, but had not been tested for MPXV.

### Clinical Features

The median incubation period was 7 days (range, 2–28 days; n = 56 with documentation of clear exposure). The most common site of onset of lesion(s) was on the genitals, reported by 74 individuals (33%). In 50 individuals (23%), lesion onset was in the perianal region or proctitis; in the rest, lesion onset was at another site (71 individuals [32%]) or lesions were disseminated over >1 region at onset (26 individuals [12%]). In 3 individuals (1%), tonsillitis was the presenting syndrome. In the 38 individuals reporting anal insertive (not receptive) sex, the majority (24 [63%]) developed their first lesions on their genitalia. In the 33 individuals reporting anal receptive (not insertive) sex, the majority (20 [61%]) developed their first lesion in the perianal region or presented with proctitis. Seven of 49 individuals (14%) reported that they did not have receptive anal sex but developed their first lesion in the perianal region or presented with proctitis.

Details pertaining to individuals’ systemic symptoms and lesion evolution are presented in Table 2. Systemic symptoms (beyond lymphadenopathy) were reported by 177 individuals (80%), with 150 individuals (68%) experiencing fevers. Fifty-five individuals (25%) experienced systemic symptoms after the onset of lesions, rather than a prodrome, and 41 individuals (19%) reported no systemic symptoms.

**Table 2.** Clinical Features of Individuals With Confirmed Mpox

Systemic Symptoms and Lesion Evolution	Individuals (N = 221)
Systemic symptoms	
Any systemic symptoms (excluding lesions or lymphadenopathy)	177 (80)
Subjective fever	150 (68)
Lymphadenopathy	130 (59)
Fatigue	89 (40)
Headache	74 (33)
Myalgia	66 (30)
Diffuse macular-papular rash	2 (1)
No systemic symptoms	41 (19)
Missing data	3 (1)
Mucosal/genitalia complications	
Lesion(s) of oral mucosa	39 (18)
Sore throat or tonsillitis	29 (13)
Perianal lesions/proctitis	90 (41)
Penile edema ± inability to retract the prepuce	17 (8)
Paraphimosis with necrotic prepuce requiring circumcision	1 (<1)
Hematuria	4 (2)
Vulval swelling	1 (<1)
Cellulitis of the genitals	1 (<1)
Urinary retention	1 (<1)
Onset of systemic symptoms	
Preceded the onset of lesion(s)	46 (21)
Concurrent to onset of lesion(s)	36 (16)
After onset of lesions(s)	55 (25)
Systemic symptoms without dermal symptoms	1 (<1)
No systemic symptoms	41 (19)
Missing data	42 (19)
Lesion evolution	
Disseminated to ≥1 additional body part	163 (74)
Localized to 1 body part/did not disseminate	58 (26)
Total No. of lesions	
Solitary lesion only	15 (7)
2–29	118 (53)
30–100	10 (5)
≥100	1 (<1)
No dermal lesions, tonsillitis alone	1 (<1)
No dermal lesions, proctitis alone	2 (1)
Missing data	74 (33)
Site of first lesion	
Torso or extremities	31 (14)
Perianal or proctitis	21 (10)
Genitals	75 (34)
Head or neck	15 (7)
Perioral or oral	37 (17)
Disseminated	32 (14)
Tonsillitis	1 (0)
No lesions	3 (1)
Missing data	6 (3)
Reason for admission	
Treatment group	
Group A: admission for clinical need	23 (10)
Cellulitis requiring intravenous antibiotics	8 (4)
Dysphagia	2 (1)
Suspected quinsy	1 (<1)
Tongue swelling with concern for airway	1 (<1)
Tongue necrosis required debridement	1 (<1)
Ophthalmic involvement	1 (<1)
Symptomatic control	1 (<1)
Paraphimosis requiring circumcision	1 (<1)
Systematically unwell with reactive arthritis	1 (<1)
Bloody diarrhea and urinary retention	1 (<1)
Pyelonephritis	1 (<1)
Psychiatric illness	1 (<1)
Gout	1 (<1)
Immunosuppression (HIV with CD4^+^ <200 cells/μL)	1 (<1)
Methicillin-susceptible *Staphylococcus aureus* bacteremia	1 (<1)
Group B: admission for social need	7 (3)
No fixed abode or unable to safely isolate	4 (2)
Diagnosis prior to introduction of virtual ward	3 (1)
Group C: not requiring admission	191 (86)
Cases of concomitant STIs (excluding previous known diagnosis of blood-borne viruses)	
STI	
*Neisseria gonorrhoeae* (gonorrhoeae)	26 (12)
*Treponema pallidum* subsp *pallidum* (syphilis)	17 (8)
*Chlamydia trachomatis* (chlamydia)	14 (6)
Herpes simplex virus 1 or 2	5 (2)
*Mycoplasma genitalium*	1 (<1)
HIV-1 (new diagnosis)	1 (<1)
Hepatitis C virus (new diagnosis)	1 (<1)

Data are presented as No. (%).

Abbreviations: HIV, human immunodeficiency virus; STI, sexually transmitted infection.

**Figure 3.**
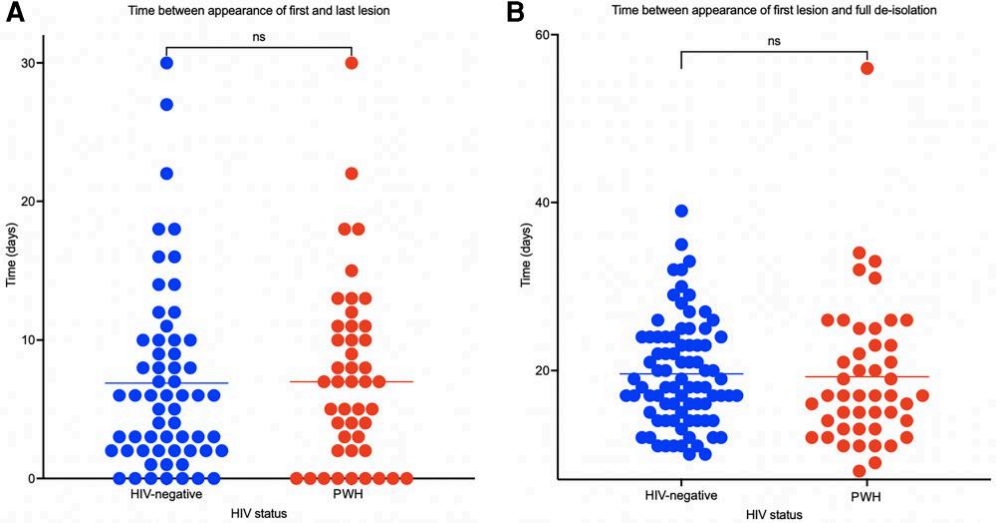
Duration between appearance of first and last mpox lesions (*A*) and appearance of first mpox lesion and full de-isolation (*B*) when comparing between human immunodeficiency (HIV)–negative individuals and people with HIV (PWH). Of the participants, 73 of 88 (82%) PWH were known to be virally suppressed on antiretroviral therapy at the point of mpox diagnosis. Abbreviations: HIV, human immunodeficiency virus; ns, not significant; PWH, people with human immunodeficiency virus.

Lesions progressed to disseminate to at least 1 additional body part in 163 (74%), and 114 (52%) individuals reported genital lesion(s) during their clinical course. The majority of PWH were known to be virally suppressed on ART on diagnosis of mpox (73/88 [82%]). There was no difference in the duration over which new lesions continued to appear between individuals who were HIV negative (n = 60/128; mean of 6.9 days) and PWH (n = 45/88; mean of 7.3 days) (2-tailed *t* test, *P* = .75; Figure 3*A*). The median time between onset of first lesion and de-isolation with complete lesion reepithelialization was 18 days (range, 8–56 days). There was no difference between time from first lesion to complete reepithelialization and de-isolation between individuals who were HIV negative (n = 82/128; mean of 19.6 days) and PWH (n = 46/88; mean of 12.3 days) (2-tailed *t* test, *P* = .79; Figure 3*B*). Only 3 individuals (1%) had clearance swabs performed at our centers to aid de-isolation.

### Management on the Virtual Ward

Most individuals were managed exclusively as outpatients (191/221 [86%]) (Table 2). No patients initially managed as an outpatient required subsequent admission. Fifty-six patients (25%) received treatment for 1 or more concomitant STIs, most frequently *Neisseria gonorrhoeae* (26 individuals [12%]) and *Treponema pallidum* subsp *pallidum* (syphilis, 17 individuals [8%]) (Table 2). Thirty-five outpatients (16%) were prescribed antibiotics for other suspected infections, predominantly for secondary cellulitis. Sixty outpatients (27%) were supplied with symptomatic treatment: analgesia (orally or topical anesthetic gels/sprays), anti-inflammatories (including topical steroids), or for constipation (stool softeners).

We noted that the majority of patients were both clinically and psychologically stable while isolating. Therefore, the frequency of assessments was reduced from every 48 hours (at the launch of the virtual ward) to once weekly by July 2022. Over the whole period, the median number of remote reviews after initial assessment was 3 (range, 0–14). Twenty-four individuals (11%) disengaged from follow-up within 4 days of being tested for MPXV testing (they were either uncontactable after testing or disengaged after being informed that their result was positive).

Forty-three individuals (19%) opted to email at least 1 photograph or set of photographs of their lesion(s) as part of their clinical review for complications or to aid de-isolation decisions.

Running the virtual ward required an additional 1.0 full-time equivalent infectious diseases specialty registrar.

### Inpatient Management

Thirty individuals (14%) required admission. Twenty-three individuals (10%) were risk group A, with clinical requirement for admission (the commonest cause of which was secondary bacterial cellulitis requiring intravenous antibiotics; see Table 2). Seven individuals (3%) were group B and admitted for isolation purposes. Three received tecovirimat and 2 received systemic steroids [25]. Most admissions were short (median duration, 4 days [range, 1–22 days]), and the majority were able to continue their isolation at home via the virtual ward.

## DISCUSSION

Our data demonstrate that 86% of patients with mpox were managed as outpatients via the virtual ward. This enabled the safe management of an evolving HCID outbreak in an outpatient setting for the first time in the UK and informed a change in National NHS England guidelines [26]. No patients initially managed as an outpatient required subsequent admission. This strategy averted a large number of hospital admissions with the requirement of 1 additional full-time specialty registrar.

The virtual ward was able to manage a range of patients including those experiencing systemic symptoms and with numerous disseminated lesions. This is in line with recent data reporting that a virtual ward can safely assess and manage mpox-associated secondary bacterial infections [27]. This builds on the current literature describing the 2022 mpox outbreak, which provides limited or no information about the practical and logistic aspects of the management of cases managed as outpatients beyond initial diagnosis [15, 16, 28–31]. Our experience highlights some of the key considerations for safe outpatient management of mpox. The UK Advisory Committee on Dangerous Pathogens removed the HCID status of the European and North American circulating clade IIb, B.1 lineage mpox in June 2022. Based on our experience and those of other UK centers, this virtual ward model was endorsed by NHS England and was subsequently widely adopted in the UK to manage mpox cases [22].

From a health system perspective, the greatest value of our strategy was facilitating the simultaneous management of a large number of mpox cases while minimizing inpatient bed-occupancy through reliable identification of the minority of patients who required admission. Our service delivered holistic care through virtual consultations and home prescription delivery including symptomatic relief, treatment for concomitant STIs, and antibiotics for suspected secondary bacterial infection of lesions.

Our data are consistent with the clinical characteristics described in other observational analyses including demonstration of an association between the type of sex and the site of the initial lesions and atypical presentations of mpox associated with the 2022 outbreak (such as an absence of systemic symptoms or systemic symptoms occurring after lesions rather than as a prodrome). Similar to other cohorts, we observed no difference in the duration of lesions between people with well-controlled HIV and HIV-negative individuals [16, 32].

Our study has a number of limitations. First, data were extracted retrospectively from electronic patient records. Although we used a standardized pro forma, some data were missing. Second, although we collected data on psychological well-being, we did not use any validated metrics so cannot formally quantify the impact of the diagnosis and isolation on patients’ well-being. Finally, our population was predominantly drawn from GBMSM, a group often well-engaged with health services and familiar with home management of other STIs. Whether a virtual ward model would be as successful for more disenfranchised populations warrants further study.

In conclusion, we demonstrate that a virtual ward can be rapidly established by specialist centers to respond to emerging health threats and that the majority of patients with mpox can be safely managed virtually. Such strategies provide a model to respond to future outbreaks.

## Notes

***Author contributions.*** E. S. and C. E. W. conceived the idea and designed the study. C. E. W., E. S., Z. C., M. S.-T., E. M., N. M., A. D. S., H. R., and A. D. W. contributed to data collection. All authors contributed to data interpretation and reviewed the results. E. S. and C. E. W. drafted the initial manuscript. All authors contributed to manuscript writing and approved the final submitted version.

***Ethics statement.*** This project meets the National Health Service definition of a service evaluation not requiring institutional review board approval. All patient data analyzed were anonymized, with patient-identifying information removed, obviating the need for patient consent.
